# Evaluation of ChatGPT as a Source of Patient-Oriented Information on Gingival Recession

**DOI:** 10.3390/healthcare14101339

**Published:** 2026-05-13

**Authors:** Serap Karakış Akcan, Gülfem Özlü Uçan, Selin Gaş, Alima Budakçı, Tuğçe Paksoy

**Affiliations:** 1 Department of Periodontology, Faculty of Dentistry, Istanbul Gelişim University, Istanbul 34310, Türkiye; 2 Department of Oral and Maxillofacial Radiology, Faculty of Dentistry, Istanbul Gelişim University, Istanbul 34310, Türkiye; gozlu@gelisim.edu.tr; 3 Department of Oral and Maxillofacial Surgery, Faculty of Dentistry, Istanbul Gelişim University, Istanbul 34310, Türkiye; sgas@gelisim.edu.tr; 4 Department of Periodontology, Hamidiye Institute of Health Sciences, University of Health Sciences, Istanbul 34668, Türkiye; 211002111@ogrenci.sbu.edu.tr; 5 Department of Periodontology, Hamidiye Faculty of Dentistry, University of Health Sciences, Istanbul 34668, Türkiye; tugce.paksoy@sbu.edu.tr

**Keywords:** gingival recession, artificial intelligence, ChatGPT, patient education, health information quality, readability

## Abstract

**Background**: Gingival recession is a common periodontal condition. With the increasing use of artificial intelligence (AI)-based chatbots, patients frequently seek online health information. However, the reliability, accuracy, and readability of AI-generated patient-oriented information on gingival recession remain unclear. **Objective**: To evaluate the quality, accuracy, and readability of ChatGPT-generated responses to patient-oriented questions related to gingival recession. **Methods**: A total of 288 patient-oriented questions were developed by an expert panel and categorized into fourteen thematic domains. Responses generated by ChatGPT (version 3.5) were independently evaluated by five oral health professionals using a modified Brief DISCERN instrument, an accuracy scoring system, and the Global Quality Score (GQS). Readability was assessed using the Flesch Reading Ease and Flesch–Kincaid Grade Level indices. **Results**: Significant differences were observed among thematic categories for DISCERN, accuracy, GQS, and readability scores (all *p* < 0.01). The highest modified Brief DISCERN, accuracy, and GQS scores were recorded for the *Information Sources/AI Reliability* category (DISCERN: 19.60 ± 2.29; accuracy: 4.67 ± 0.49; GQS: 4.33 ± 0.49), whereas the lowest scores were observed for the *What Happens If Left Untreated?* category (DISCERN: 14.27 ± 1.75; accuracy: 3.23 ± 0.43). Strong positive correlations were identified between DISCERN and accuracy (*r* = 0.784, *p* < 0.001) and between accuracy and GQS (*r* = 0.868, *p* < 0.001). Readability indices were not significantly correlated with accuracy or quality measures. **Conclusions**: ChatGPT provided patient-oriented information on gingival recession with variable performance across thematic domains; however, readability remained a limitation. AI-generated content should therefore be considered a supplementary resource rather than a substitute for clinician-guided patient communication.

## 1. Introduction

Gingival recession is commonly defined as the apical displacement of the gingival margin with subsequent root surface exposure and is a frequent clinical finding in the general population [1,2,3,4]. Recession-associated attachment loss is most often observed on buccal tooth surfaces and may occur even in individuals with high oral hygiene standards. Although mechanical factors such as traumatic tooth brushing and orthodontic movement have been suggested as contributing factors, the causal relationships remain complex and not fully established [2,4]. Root surface exposure may be accompanied by dentin hypersensitivity, root caries, non-carious cervical lesions, plaque control difficulties, and esthetic concerns, which together can adversely affect patient comfort and daily oral hygiene practices [5,6,7]. Longitudinal observations indicate that untreated buccal gingival recessions may remain stable in some individuals but may progress in others, underscoring the importance of individualized risk assessment rather than uniform assumptions regarding disease course [6,8,9].

Surgical intervention may be considered in the presence of esthetic concerns, dentin hypersensitivity, signs of active or progressive recession, or when orthodontic or restorative treatment is planned for teeth with predisposing local factors, such as inadequate keratinized tissue or unfavorable anatomical conditions [10,11,12,13]. Periodontal plastic surgery techniques, including pedicle flaps and free gingival grafts, are commonly employed to address recession-related problems, while connective tissue grafts combined with coronally or laterally advanced flaps are frequently discussed when root coverage and esthetic outcomes are primary treatment goals [7,13,14]. Nevertheless, not all patients are willing to undergo surgical root coverage procedures. Treatment planning and communication should therefore take into account both site-related and patient-related factors, including expectations, tolerance for postoperative discomfort, and perceived trade-offs between esthetic benefit and morbidity [6,13,14]. Patient-reported burdens such as postoperative pain, bleeding, and functional limitations have been documented following mucogingival surgery and may influence treatment acceptance and satisfaction [7,15]. Accordingly, the incorporation of patient-reported outcomes into periodontal research and clinical communication has gained increasing emphasis [16].

Artificial intelligence (AI) technologies are being progressively integrated into healthcare, including dentistry, where they are increasingly being explored as decision-support and educational tools [17]. During periodontal consultations, particularly when surgical interventions are discussed, patients often seek additional information regarding disease progression, treatment options, expected outcomes, and postoperative experiences [7,16]. This information-seeking behavior may contribute to uncertainty, anxiety, and delayed decision-making when professional guidance is not readily available [18,19,20]. In parallel, patients increasingly rely on online sources for health-related information, and large language model-based chatbots such as ChatGPT have introduced a novel, conversational modality for information retrieval [21,22]. While these systems may enhance accessibility and engagement, concerns persist regarding the reliability, completeness, and clinical appropriateness of AI-generated health information, particularly in conditions requiring individualized assessment and nuanced decision-making [20,21,22].

Although ChatGPT has been evaluated in several dental and surgical contexts, evidence specifically addressing patient-oriented information related to gingival recession remains limited [18,19,23,24]. In particular, the quality, factual accuracy, and readability of ChatGPT-generated responses have not been comprehensively examined across clinically relevant domains of gingival recession using a category-based, patient-oriented framework. Given that gingival recession encompasses both general informational concerns and context-dependent treatment communication, it provides a clinically relevant model for evaluating how AI-generated responses perform across different types of patient information needs. Therefore, the aim of the present study was to evaluate the quality, accuracy, and readability of ChatGPT-generated responses to patient-oriented questions on gingival recession across predefined clinically relevant thematic categories.

## 2. Materials and Methods

### 2.1. Question Selection and Preparation

Patient-oriented questions related to gingival recession were developed using a structured, theory-informed approach designed to reflect common information-seeking behaviors encountered in periodontal clinical practice. The question development process was informed by established periodontal diagnostic frameworks, gingival recession classification systems, and widely accepted principles of mucogingival therapy, prosthesis–periodontium interactions, and patient-centered care, as described in standard periodontology textbooks and contemporary clinical guidelines [25]. Particular emphasis was placed on addressing both the biological and psychosocial dimensions of gingival recession to ensure a comprehensive representation of clinically relevant patient concerns.

An expert panel consisting of three periodontists (two with over ten years of clinical practice and one periodontology PhD candidate), one oral and maxillofacial surgeon, and one oral and maxillofacial radiologist defined fourteen thematic categories *a priori* to guide question development and ensure balanced coverage of key domains. These categories were: *Definition/General Information, Causes/Risk Factors, Prevention/Protective Measures, Symptoms/Daily Life Impacts, Treatment Options, Suitable/Not Suitable, Complications/Success Rates, Preprosthetic Period, Postprosthetic Period, Early Post-op (0–7 days), Late Post-op (1 week–6 months), What Happens If Left Untreated?, Psychological/Social Effects, and Information Sources/AI Reliability* (Table 1) [1,2,4,5,6,7,8,9,11,12,13,14,17,19,21,23,25,26,27,28,29,30,31,32,33,34,35]. The categorization strategy was grounded in clinical reasoning frameworks rather than data-driven clustering in order to preserve interpretability and relevance to routine periodontal practice.

Questions were formulated in clear, patient-centered language to simulate real-world inquiries commonly raised during chairside consultations and online health information searches. During the drafting phase, an artificial intelligence-based language model was used solely as an editorial support tool to enhance linguistic clarity and variability of phrasing. The conceptual content, clinical scope, and final inclusion of all questions were determined exclusively by the expert panel, and no AI-generated content was used to define question topics, clinical assumptions, or thematic structure.

A total of 288 unique questions were finalized. All questions were independently reviewed by two authors to identify and eliminate duplicate or semantically overlapping items. Minor linguistic refinements were applied where necessary without altering the original intent or clinical meaning of the questions. All questions were originally developed in Turkish and subsequently translated into English prior to querying ChatGPT. The translations were reviewed by two bilingual authors to ensure semantic accuracy and patient-oriented clarity. The complete list of finalized questions, organized according to thematic category, is provided in Supplementary File S1.

### 2.2. AI Response Generation

All finalized questions were submitted to ChatGPT (OpenAI, San Francisco, CA, USA; https://chat.openai.com/, accessed on 12 June 2025) using a publicly accessible, non-subscribed user interface based on the GPT-3.5 model. Each question was entered independently into a new conversation window in order to prevent contextual carryover between responses and to approximate real-world patient use conditions. No follow-up prompts, clarifications, or iterative refinements were applied. All responses were recorded verbatim and stored for subsequent evaluation.

### 2.3. Evaluation of AI-Generated Responses

All AI-generated responses were independently evaluated by five assessors (three periodontists, one oral and maxillofacial surgeon, and one oral and maxillofacial radiologist), none of whom were involved in question development, data analysis, manuscript development, or AI response generation. Assessors were blinded to each other’s ratings. Before the formal evaluation, all assessors were provided with predefined scoring criteria and item-level definitions. These criteria were reviewed jointly in a calibration step to promote a consistent interpretation of the assessment framework prior to independent blinded scoring. Evaluations focused exclusively on content accuracy, balance, and patient-oriented usefulness; stylistic preferences, tone, and response length were not considered.

### 2.4. Brief Discern Assessment

The quality and reliability of the AI-generated responses were assessed using an adapted five-item version of the Brief DISCERN instrument. The original Brief DISCERN, developed by Khazaal et al. (2009), is a validated tool derived from the original DISCERN and designed to evaluate the quality of evidence-based patient health information [36]. In the original Brief DISCERN, six items were retained to represent the principal evaluative domains identified during item reduction and psychometric testing.

In the present study, a shortened five-item derivative was employed to allow practical and consistent evaluation of a large number of brief AI-generated responses while preserving the principal conceptual domains of the Brief DISCERN framework. The retained items addressed source reliability, currency of information, balanced and unbiased presentation of treatment-related content, communication of benefits and risks, and support for informed patient decision-making, which are also reflected in the detailed scoring criteria provided in Supplementary Table S1. The quality-of-life-related domain represented in the original six-item Brief DISCERN was not retained as a separate item because this aspect was infrequently and inconsistently addressed in short chatbot responses and was considered likely to reduce scoring consistency in the present response format. Accordingly, this adapted form should be interpreted as a study-specific operational derivative rather than as a newly validated replacement for the original Brief DISCERN instrument. Each item was rated on a five-point Likert scale (1 = not present, 5 = completely fulfilled), and total DISCERN scores were calculated by summing the individual item scores. Detailed item definitions and scoring criteria are provided in Supplementary Table S1. The internal consistency of the modified five-item Brief DISCERN form was assessed using Cronbach’s alpha (α = 0.840).

### 2.5. Accuracy and Global Quality Assessment

Accuracy was evaluated using a five-point Likert scale previously described in the literature, ranging from completely incorrect information (score 1) to completely correct information (score 5) [19,37]. Accuracy ratings were assigned by the independent assessors with reference to the current periodontal literature, established clinical knowledge, and the thematic reference framework used for question development and categorization (Table 1), rather than against a single prewritten benchmark answer. Overall informational quality was assessed using the standard five-point Global Quality Score (GQS), which evaluates the completeness, clarity, accuracy, and usefulness of health-related information for patients [38].

### 2.6. Readability Assessment

Readability was evaluated using the Flesch Reading Ease (FRE) and Flesch–Kincaid Grade Level (FKGL) indices, calculated automatically using a standardized online readability analysis tool (Readable Pro; https://app.readable.com/text/, accessed on 25 July 2025). FRE scores range from 0 to 100, with lower scores indicating increased textual complexity, while FKGL scores estimate the number of years of formal education required to comprehend the text. In line with commonly cited recommendations for patient education materials, an FKGL score of sixth grade or below was considered appropriate for patient-oriented health information [23,39,40].

**Table 1:** Thematic categories used for patient-oriented questions related to gingival recession.

Category No.	Thematic Category	Scope/Rationale	Reference(s) *
1	Definition/General Information	Basic concepts and classification	[1,2,4,27,28]
2	Causes/Risk Factors	Etiology and risk factors	[1,2,4,8]
3	Prevention/Protective Measures	Prevention and protective advice	[2,6]
4	Symptoms/Daily Life Impacts	Symptoms and daily-life effects	[7,16,32,33,34,35]
5	Treatment Options	Surgical and non-surgical options	[13,14,26]
6	Suitable/Not Suitable	Indications and case selection	[6,10,14]
7	Complications/Success Rates	Complications and predictability	[8,15]
8	Preprosthetic Period	Before crown placement or prosthetic treatment	[11,29,31]
9	Postprosthetic Period	Existing prostheses, crown margins, and grafted tissues	[29,30,31]
10	Early Post-op (0–7 days)	Early healing and short-term care	[14,15]
11	Late Post-op (1 week–6 months)	Later healing and recovery	[9,15,35]
12	What Happens If Left Untreated?	Natural course and consequences	[8]
13	Psychological/Social Effects	Psychosocial and esthetic impacts	[32,33,34,35]
14	Information Sources/AI Reliability	Information pathways and AI reliability	[17,19,20,21,22,23,24,41]

* Reference numbers correspond to the main reference list and indicate the conceptual framework used for thematic categorization.

### 2.7. Inter-Rater Reliability

Inter-rater reliability among the five independent assessors was evaluated for the rater-based outcome measures, including the modified Brief DISCERN score, accuracy ratings, and the Global Quality Score (GQS), using the intraclass correlation coefficient (ICC) based on a two-way random-effects model with absolute agreement. ICC values and 95% confidence intervals were reported (Table 2).

Readability indices (FRE and FKGL) were calculated automatically; therefore, inter-rater reliability analysis was not applicable to these measures.

### 2.8. Statistical Analysis

Descriptive statistics, including frequency, percentage, mean, standard deviation, median, and interquartile range, were calculated for all outcome measures. Normality was assessed using the Shapiro–Wilk test. Comparisons among thematic categories were performed using the Kruskal–Wallis test for non-normally distributed variables, followed by Bonferroni-corrected post hoc analyses where appropriate. Correlations between continuous variables were evaluated using Pearson correlation analysis for normally distributed variables and Spearman’s rank correlation analysis for non-normally distributed or ordinal variables. Effect sizes for between-category comparisons were estimated using eta-squared (η^2^), and 95% confidence intervals were reported. All statistical analyses were conducted using IBM SPSS Statistics version 27.

## 3. Results

The modified five-item Brief DISCERN form showed a Cronbach’s alpha value of 0.840 in the present sample. In addition, inter-rater reliability among the five independent assessors was high across all evaluator-based outcome measures. The intraclass correlation coefficient (ICC) for the modified Brief DISCERN score was 0.901 (95% CI, 0.881 to 0.916), compared with 0.843 (95% CI, 0.805 to 0.877) for accuracy and 0.853 (95% CI, 0.818 to 0.887) for the Global Quality Score (GQS). These findings indicate strong inter-rater agreement among the assessors (Table 2).

**Figure 1:**
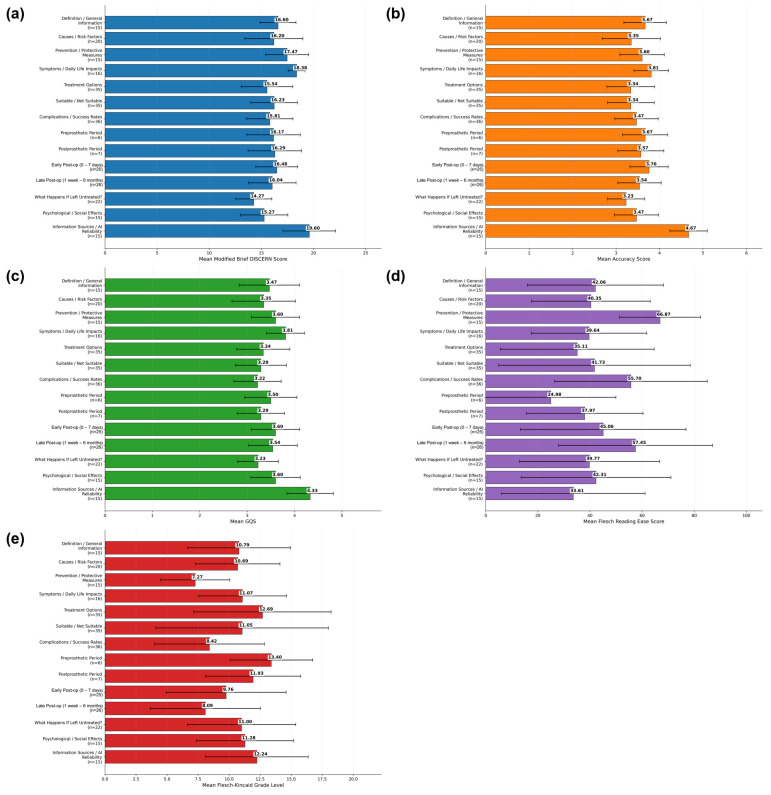
Category-based mean scores across gingival recession question categories. (**a**) Modified Brief DISCERN; (**b**) Accuracy; (**c**) Global Quality Score (GQS); (**d**) Flesch Reading Ease; (**e**) Flesch–Kincaid Grade Level. Error bars indicate standard deviations.

Descriptive statistics for the overall modified Brief DISCERN, accuracy, GQS, and readability scores across all responses, irrespective of thematic category, are presented in Table 3. The mean modified Brief DISCERN score was 16.27 ± 2.39, while the mean accuracy and GQS scores were 3.55 ± 0.59 and 3.47 ± 0.57, respectively. Regarding readability, the mean Flesch Reading Ease score was 44.51 ± 29.39, and the mean Flesch–Kincaid Grade Level score was 10.45 ± 4.82. Category-specific mean scores are presented in Figure 1a–e. Effect size estimates for the overall outcome measures ranged from small to moderate, with the highest eta-squared values observed for accuracy (*η*^2^ = 0.278) and modified Brief DISCERN (*η*^2^ = 0.240) (Table 3).

### 3.1. Discern Scores by Question Category

The distribution of modified Brief DISCERN scores across question categories is presented in Table 4. Statistically significant differences were observed among categories (Kruskal–Wallis test, *p* < 0.001).

The highest mean DISCERN scores were recorded for *Information Sources/AI Reliability* (19.60 ± 2.29), followed by *Symptoms/Daily Life Impacts* (18.38 ± 0.81). In contrast, the lowest scores were observed for *What Happens If Left Untreated?* (14.27 ± 1.75).

Post hoc Bonferroni analyses demonstrated that the *What Happens If Left Untreated?* category exhibited significantly lower DISCERN scores compared with *Prevention/Protective Measures, Symptoms/Daily Life Impacts*, and *Information Sources/AI Reliability* (all *p* < 0.01). In addition, *Treatment Options, Complications/Success Rates, Psychological/Social Effects*, and *Suitable/Not Suitable* showed significantly lower scores than *Symptoms/Daily Life Impacts* and *Information Sources/AI Reliability*. Detailed pairwise comparisons are presented in Supplementary Table S2. The category-specific mean modified Brief DISCERN scores are shown in Figure 1a.

### 3.2. Accuracy Scores by Question Category

Accuracy scores differed significantly across question categories (Kruskal–Wallis test, *p* < 0.001; Table 5). The highest accuracy scores were observed for *Information Sources/AI Reliability* (4.67 ± 0.49), whereas lower scores were noted for *What Happens If Left Untreated?* (3.23 ± 0.43), Treatment Options (3.34 ± 0.54), and *Suitable/Not Suitable* (3.34 ± 0.54).

Post hoc analyses indicated that all categories—except *Symptoms/Daily Life Impact*s, *Preprosthetic Period*, and *Postprosthetic Period*—had significantly lower accuracy scores compared with *Information Sources/AI Reliability*. The category-specific mean accuracy scores are shown in Figure 1b.

### 3.3. Global Quality Score (Gqs)

The distribution of the Global Quality Score (GQS) across question categories is presented in Table 6. Statistically significant differences were observed among categories (Kruskal–Wallis test, *p* < 0.001).

The highest GQS values were recorded for *Information Sources/AI Reliability* (4.33 ± 0.49), whereas lower scores were observed for *Complications/Success Rates* (3.22 ± 0.48) and *What Happens If Left Untreated?* (3.23 ± 0.43). Post hoc analyses showed that *Treatment Options, Causes/Risk Factors, Complications/Success Rates, Suitable/Not Suitable*, and *Late Post-op (1 week–6 months)* had significantly lower GQS scores compared with *Information Sources/AI Reliability.* Additionally, *Complications/Success Rates* demonstrated significantly lower GQS scores than *Symptoms/Daily Life Impacts*. Detailed comparisons are provided in Supplementary Table S4. The category-specific mean GQS scores are shown in Figure 1c.

### 3.4. Readability Analysis

Significant differences in Flesch Reading Ease (FRE) scores were observed among question categories (Kruskal–Wallis test, *p* = 0.007; Table 7). The highest readability scores were observed for *Prevention/Protective Measures*, whereas lower FRE scores were noted for *Preprosthetic Period* and *Treatment Options*. The category-specific mean Flesch Reading Ease scores are shown in Figure 1d.

Post hoc analysis identified a significant difference between *Treatment Options* and *Prevention/Protective Measures*, with lower readability observed in the former (Supplementary Table S5).

Flesch–Kincaid Grade Level (FKGL) scores also differed significantly across categories (Kruskal–Wallis test, *p* = 0.001; Table 8). Higher FKGL values, indicating greater textual complexity, were observed for *Treatment Options* and *Preprosthetic Period*. Detailed post hoc comparisons are presented in Supplementary Table S6. The category-specific mean Flesch–Kincaid Grade Level scores are shown in Figure 1e.

### 3.5. Correlation Analyses

Overall correlations between evaluation metrics are presented in Table 9. Strong positive correlations were observed between DISCERN and accuracy scores (r = 0.784), DISCERN and GQS (r = 0.769), and accuracy and GQS (r = 0.868) (all *p* < 0.001). A very strong inverse correlation was identified between FRE and FKGL scores (r = –0.972, *p* < 0.001).

Category-specific correlation analyses are summarized in Supplementary Table S7.

## 4. Discussion

Accurate and comprehensible patient information represents an important component of periodontal care, particularly in conditions such as gingival recession, where treatment decisions are influenced not only by clinical findings but also by patient perception, esthetic expectations, and anticipated long-term outcomes. Gingival recession is a biologically heterogeneous condition characterized by variability in etiology, progression patterns, and treatment indications, which complicates the standardization of patient education strategies [4,6,8]. Within this context, patients increasingly seek preliminary or Supplementary Information from online sources, either before or alongside professional consultations. Recent investigations in dentistry and medicine have shown that large language model-based systems, including ChatGPT, are often capable of providing useful descriptive health information. However, their performance appears more variable when addressing clinically complex, decision-dependent, or individualized topics [18,19,21]. Consistent with this emerging evidence, the present study demonstrated that ChatGPT-generated responses to patient-oriented questions on gingival recession varied across thematic categories. Higher evaluation scores were observed in domains related to *Symptoms/Daily Life Impacts* and *Information Sources/AI Reliability*, whereas comparatively lower scores were noted in categories involving *What Happens If Left Untreated?, Treatment Options*, and *Complications/Success Rates*. Taken together, these findings suggest that ChatGPT may function more effectively as a general informational resource than as a tool capable of conveying nuanced, context-dependent periodontal decision-making.

Clinical decision-making in the management of gingival recession is multifactorial and context-dependent. Treatment indications are influenced by recession classification, periodontal phenotype, anatomical constraints, esthetic demands, patient expectations, and the presence of planned orthodontic or restorative interventions [6,7,10,13]. Moreover, longitudinal evidence suggests that untreated buccal gingival recessions may remain stable in some individuals but may progress in others, underscoring the absence of a universal treatment threshold and the need for individualized risk assessment [8,9]. In the present study, categories related to *Treatment Options, Complications/Success Rates*, and *What Happens If Left Untreated*? consistently demonstrated lower modified Brief DISCERN, accuracy, and GQS scores than more general informational domains. Similar patterns have been reported in previous evaluations of AI-based patient information, in which language models showed reduced consistency when addressing treatment indications, prognostic uncertainty, or long-term outcomes rather than descriptive disease-related content [19,21,41]. This pattern may be explained by the inherent complexity of periodontal treatment decision-making. In these domains, ChatGPT responses frequently adopted generalized and cautious language, emphasizing clinical variability and the need for professional evaluation. While such caution may reduce the risk of explicit misinformation, it may also result in responses that lack sufficient specificity when judged against established clinical standards and evidence-based frameworks [6,7,13]. Accordingly, the comparatively lower performance observed in these categories likely reflects the individualized and context-sensitive nature of gingival recession management rather than a broader inability of AI-generated patient information to provide useful support.

In the contemporary digital health landscape, questions addressing information sources and the reliability of health-related content form a distinct domain when compared with clinically oriented diagnostic or therapeutic inquiries. Such questions primarily focus on the credibility of information, the limitations of non-professional sources, and the appropriate role of clinician consultation, rather than on individualized treatment decisions. Previous research in both dentistry and medicine has shown that large language models tend to perform more favorably in these guidance-oriented contexts, where responses are expected to emphasize caution, transparency, and referral to professional care [19,20,21,23]. In the present study, the *Information Sources/AI Reliability* category achieved the highest scores across DISCERN, accuracy, and Global Quality Score assessments. This finding is consistent with earlier evaluations of AI-generated patient information, which have shown that responses explicitly acknowledging uncertainty, advising professional verification, and discouraging self-diagnosis are more likely to be rated as reliable and balanced [19,20]. Importantly, this favorable performance should not be interpreted as evidence of superior clinical reasoning. Rather, it suggests that AI systems may be better suited to guidance-focused questions than to condition-specific clinical judgments. From a patient education perspective, this distinction supports the view that AI-generated content may serve as an adjunct to, rather than a replacement for, professional periodontal consultation.

Previous studies in dentistry and periodontology suggest that the performance of AI-generated patient information varies according to both the clinical topic and the model evaluated [23,37,41,42]. In periodontology, ChatGPT has been reported to provide generally accurate and sufficiently complete answers to frequently asked patient questions, although expert supervision remains necessary because of the risk of incomplete or potentially misleading information [42]. Comparative studies have also shown measurable differences among ChatGPT-3.5, ChatGPT-4, Gemini, and Copilot in terms of quality, reliability, readability, and comprehensiveness [23,37,41]. Model-dependent variability has also been demonstrated in periodontology-focused educational and question-answering settings, where newer or alternative systems have outperformed earlier ChatGPT versions on certain tasks [43,44]. In addition, broader evidence suggests that the availability of newer models does not eliminate important concerns regarding readability, specificity, and the overall limitations of AI-mediated patient information in patient education settings [45]. Accordingly, the present findings should not be interpreted as representing the performance of AI chatbots in general, nor as a stable benchmark over time. Rather, they are best understood as a contextual evaluation of a specific publicly accessible model at a specific stage in the evolution of generative AI. GPT-3.5 was selected because it was publicly accessible and widely used at the time of data collection [19], thereby reflecting a realistic patient-facing use scenario. Because chatbot performance may vary across models and over time, the present findings are best interpreted as model- and time-specific rather than as temporally stable properties of newer AI systems.

Readability constitutes a critical but often underestimated component of patient education, particularly in periodontal care, where understanding disease mechanisms, treatment options, and postoperative expectations can directly influence adherence and decision-making. Previous research has shown that health information may be technically accurate and evidence-based, yet remain functionally ineffective if it exceeds patients’ reading and comprehension levels [32,33,34]. This distinction is especially relevant for digital and AI-generated content, in which linguistic complexity may increase as informational detail expands [21]. In the present study, readability metrics varied significantly across thematic categories, with higher Flesch–Kincaid Grade Level scores observed in domains characterized by dense clinical content, such as *Treatment Options* and *Preprosthetic Period*. Similar patterns have been reported in prior evaluations of ChatGPT and other digital health resources, where acceptable accuracy and quality scores frequently coexisted with reading levels exceeding recommended thresholds for patient-oriented materials [39,40,46]. These findings suggest that AI-generated responses may preserve informational detail at the expense of linguistic accessibility. Accordingly, even balanced and factually appropriate responses may remain difficult for patients to understand without professional guidance, reinforcing the need for clinician involvement in contextualizing and simplifying AI-provided information.

The strong positive correlations among modified Brief DISCERN, accuracy, and Global Quality Score indicate substantial internal coherence among the evaluator-based measures used in this study. This is expected, as these instruments overlap in their emphasis on factual correctness, balance of information, and support for informed decision-making. Previous studies of online health information and AI-generated medical content have also shown that materials perceived as more reliable tend to receive higher accuracy and overall quality ratings [19,39,46]. In the present analysis, the particularly strong association between accuracy and GQS suggests that factual correctness played a major role in global quality judgments, consistent with prior AI-focused evaluations in dentistry and medicine [18,21]. The observed correlations between DISCERN and both accuracy and GQS further support the alignment of the modified Brief DISCERN approach with the other evaluator-based measures. In contrast, the absence of meaningful correlations between readability indices and evaluator-based metrics suggests that linguistic accessibility represents a distinct dimension of health communication. Similar dissociations have been reported in studies of digital patient education materials, in which higher informational quality or accuracy did not necessarily correspond to better readability or patient comprehension [46,47,48]. Taken together, these findings indicate that reliability, accuracy, and global quality moved in parallel in the present sample, whereas readability reflected a separate dimension of AI-generated patient information.

Several limitations should be considered when interpreting the present findings. First, the question set was developed within an expert-framed, category-based structure designed to ensure thematic coverage and standardized comparison; however, it may not fully reflect the spontaneity, wording, and priorities of real-world patient inquiries. Second, only GPT-3.5 was evaluated; because chatbot performance may vary across models and over time, the present findings should be interpreted as model- and time-specific rather than as temporally stable properties of newer AI systems [20,21,43,44]. Third, the responses were evaluated by oral health professionals rather than by patients and therefore do not directly capture how individuals with different levels of health literacy, prior knowledge, or digital experience may understand and use the same information [46,47]. Finally, although the selected tools provided structured and clinically relevant assessments, they were originally developed for written health information and may not fully capture all dimensions of AI-generated communication, including conversational nuance and the effect of follow-up questioning [19,36,39]. Accordingly, future studies should incorporate authentic patient-generated questions, patient-based evaluations, multi-model comparisons, and more naturalistic multi-turn interactions.

## 5. Conclusions

This study evaluated ChatGPT as a patient-oriented information source for gingival recession across fourteen clinically relevant categories. Performance varied according to topic complexity, with stronger results in general and guidance-oriented domains and lower scores in categories requiring more individualized clinical judgment. Readability remained a notable limitation and appeared to be a distinct challenge independent of informational accuracy or global quality. Taken together, these findings suggest that ChatGPT may serve as a supplementary informational resource for patient education in periodontology, but should not replace clinician–patient communication in contexts requiring individualized assessment, clinical judgment, and shared decision-making. Future studies should incorporate real patient-generated questions, broader participant perspectives, and direct comparisons across newer chatbot models.

Clinical Significance: Artificial intelligence-generated information may support patient awareness and general understanding of gingival recession. However, variability in content quality and limitations in readability emphasize the continued need for clinician-guided interpretation, particularly in treatment-related discussions. AI-based tools should be used as adjunctive resources rather than replacements for professional clinical communication.

## Figures and Tables

**Table 2:** Inter-rater reliability of evaluator-based outcome measures.

Outcome Measure	ICC	95% CI
Modified Brief DISCERN score	0.901	0.881 to 0.916
Accuracy	0.843	0.805 to 0.877
Global Quality Score (GQS)	0.853	0.818 to 0.887

**Abbreviations**: ICC, intraclass correlation coefficient; CI, confidence interval; GQS, Global Quality Score.

**Table 3:** Overall descriptive statistics and effect sizes for Modified Brief DISCERN, Accuracy, GQS, and readability scores.

Measure	n	Mean ± SD	Min–Max	Eta-Squared (η^2^) *	95% CI Lower	95% CI Upper
Modified Brief DISCERN	288	16.27 ± 2.39	11.0–23.0	0.240	0.125	0.289
Accuracy	288	3.55 ± 0.59	2.0–5.0	0.278	0.161	0.329
Global Quality Score (GQS)	288	3.47 ± 0.57	2.0–5.0	0.212	0.099	0.259
Flesch Reading Ease	288	44.51 ± 29.39	0.0–100.0	0.103	0.011	0.133
Flesch-Kincaid Grade Level	288	10.45 ± 4.82	0.0–26.5	0.118	0.022	0.151

**Abbreviations**: GQS, Global Quality Score; SD, standard deviation; CI, confidence interval; η^2^, eta-squared. **Footnote**: Higher modified Brief DISCERN, Accuracy, and GQS scores indicate better performance. Higher Flesch Reading Ease scores indicate easier readability, whereas higher Flesch–Kincaid Grade Level scores indicate greater reading difficulty. * Effect size estimates (η^2^) are reported for the overall category-based comparisons.

**Table 4:** Distribution and comparison of Modified Brief DISCERN scores across question categories.

	Mean ± SD	Median (Q1–Q3)	Test Statistic	*p* Value
Definition/General Information	16.60 ± 1.72	17 (16–18)	65.421	<0.001 *
Causes/Risk Factors	16.20 ± 2.78	17 (14–18.5)		
Prevention/Protective Measures	17.47 ± 2.07	18 (15–19)		
Symptoms/Daily Life Impacts	18.38 ± 0.81	18 (18–18)		
Treatment Options	15.54 ± 2.49	15 (13–17)		
Suitable/Not Suitable	16.23 ± 2.12	16 (15–17)		
Complications/Success Rates	15.81 ± 2.15	16 (13.5–18)		
Preprosthetic Period	16.17 ± 2.56	17 (13–18)		
Postprosthetic Period	16.29 ± 2.75	17 (13–19)		
Early Post-op (0–7 days)	16.48 ± 1.96	17 (16–18)		
Late Post-op (1 week–6 months)	16.04 ± 2.27	17 (14–18)		
What happens If Left Untreated?	14.27 ± 1.75	13 (13–16)		
Psychological/Social Effects	15.27 ± 1.22	16 (14–16)		
Information Sources/AI Reliability	19.60 ± 2.29	20 (17–22)		

* Kruskal–Wallis test. *p* < 0.05 was considered statistically significant. Abbreviations: SD, standard deviation; Q1–Q3, interquartile range.

**Table 5:** Distribution and comparison of Accuracy scores across question categories.

	Mean ± SD	Median (Q1–Q3)	Test Statistic	*p* Value
Definition/General Information	3.67 ± 0.49	4 (3–4)	63.008	<0.001 *
Causes/Risk Factors	3.35 ± 0.67	3 (3–4)		
Prevention/Protective Measures	3.60 ± 0.51	4 (3–4)		
Symptoms/Daily Life Impacts	3.81 ± 0.40	4 (4–4)		
Treatment Options	3.34 ± 0.54	3 (3–4)		
Suitable/Not Suitable	3.34 ± 0.54	3 (3–4)		
Complications/Success Rates	3.47 ± 0.51	3 (3–4)		
Preprosthetic Period	3.67 ± 0.52	4 (3–4)		
Postprosthetic Period	3.57 ± 0.53	4 (3–4)		
Early Post-op (0–7 days)	3.76 ± 0.44	4 (4–4)		
Late Post-op (1 week–6 months)	3.54 ± 0.51	4 (3–4)		
What Happens If Left Untreated?	3.23 ± 0.43	3 (3–3)		
Psychological/Social Effects	3.47 ± 0.52	3 (3–4)		
Information Sources/AI Reliability	4.67 ± 0.49	5 (4–5)		

* Kruskal–Wallis test. *p* < 0.05 was considered statistically significant. Abbreviations: SD, standard deviation; Q1–Q3, interquartile range.

**Table 6:** Distribution and comparison of Global Quality Score (GQS) scores across question categories.

	Mean ± SD	Median (Q1–Q3)	Test Statistic	*p* Value
Definition/General Information	3.47 ± 0.64	4 (3–4)	54.540	<0.001 *
Causes/Risk Factors	3.35 ± 0.67	3 (3–4)		
Prevention/Protective Measures	3.6 ± 0.51	4 (3–4)		
Symptoms/Daily Life Impacts	3.81 ± 0.4	4 (4–4)		
Treatment Options	3.34 ± 0.54	3 (3–4)		
Suitable/Not Suitable	3.29 ± 0.52	3 (3–4)		
Complications/Success Rates	3.22 ± 0.48	3 (3–3.5)		
Preprosthetic Period	3.5 ± 0.55	3.5 (3–4)		
Postprosthetic Period	3.29 ± 0.49	3 (3–4)		
Early Post-op (0–7 days)	3.6 ± 0.5	4 (3–4)		
Late Post-op (1 week–6 months)	3.54 ± 0.51	4 (3–4)		
What Happens If Left Untreated?	3.23 ± 0.43	3 (3–3)		
Psychological/Social Effects	3.6 ± 0.51	4 (3–4)		
Information Sources/AI Reliability	4.33 ± 0.49	4 (4–5)		

* Kruskal–Wallis test. *p* < 0.05 was considered statistically significant. Abbreviations: GQS, Global Quality Scale; SD, standard deviation; Q1–Q3, interquartile range.

**Table 7:** Distribution and comparison of Flesch Reading Ease scores across question categories.

	Mean ± SD	Median (Q1–Q3)	Test Statistic	*p* Value
Definition/General Information	42.06 ± 26.13	45.8 (21.4–58.8)	28.868	0.007 *
Causes/Risk Factors	40.35 ± 22.78	45.45 (20.95–59.3)		
Prevention/Protective Measures	66.87 ± 15.6	68.3 (60.3–78.2)		
Symptoms/Daily Life Impacts	39.64 ± 22.15	39.15 (25.25–54.25)		
Treatment Options	35.11 ± 29.77	35.9 (0.1–56.3)		
Suitable/Not Suitable	41.73 ± 35.65	43.4 (6.2–66.7)		
Complications/Success Rates	55.7 ± 29.15	52.9 (36.45–84.7)		
Preprosthetic Period	24.98 ± 24.39	20.2 (7.5–38.3)		
Postprosthetic Period	37.97 ± 22.13	37.3 (33.9–45.9)		
Early Post-op (0–7 days)	45.06 ± 31.52	36.3 (22.1–66.7)		
Late Post-op (1 week–6 months)	57.45 ± 29.45	52.05 (35.9–88.1)		
What Happens If Left Untreated?	39.77 ± 26.74	32.9 (24.4–57.3)		
Psychological/Social Effects	42.31 ± 28.9	38.3 (11.9–68.1)		
Information Sources/AI Reliability	33.61 ± 28.08	41.4 (0.1–53.9)		

* Kruskal–Wallis test. *p* < 0.05 was considered statistically significant. Abbreviations: SD, standard deviation; Q1–Q3, interquartile range.

**Table 8:** Distribution and comparison of Flesch–Kincaid Grade Level scores across question categories.

	Mean ± SD	Median (Q1–Q3)	Test Statistic	*p* Value
Definition/General Information	10.79 ± 4.16	11.1 (8.8–14.2)	33.735	0.001 *
Causes/Risk Factors	10.69 ± 3.41	10.05 (7.7–13.35)		
Prevention/Protective Measures	7.27 ± 2.79	6.8 (4.8–9.5)		
Symptoms/Daily Life Impacts	11.07 ± 3.55	11 (8.8–12.55)		
Treatment Options	12.69 ± 5.55	11.9 (9.1–17.8)		
Suitable/Not Suitable	11.05 ± 6.45	9.9 (6–15.4)		
Complications/Success Rates	8.42 ± 4.4	8.95 (4.8–10.5)		
Preprosthetic Period	13.4 ± 3.27	13.65 (11.9–15.4)		
Postprosthetic Period	11.93 ± 3.82	11.5 (9–12.3)		
Early Post-op Period (0–7 days)	9.76 ± 4.75	11.1 (7.2–12.7)		
Late Post-op Period (1 week–6 months)	8.09 ± 4.39	9.35 (4–11.1)		
What Happens If Left Untreated?	11 ± 4.29	11.5 (8.4–13.1)		
Psychological/Social Effects	11.28 ± 3.81	11.5 (8.7–15.4)		
Information Sources/AI Reliability	12.24 ± 4.13	11.1 (8–16.2)		

* Kruskal–Wallis test. *p* < 0.05 was considered statistically significant. Abbreviations: SD, standard deviation;. Q1–Q3, interquartile range.

**Table 9:** Correlation analysis between evaluation scores for AI-generated responses.

		Accuracy	GQS	Flesch Reading Ease	Flesch–Kincaid Grade Level
Modified Brief DISCERN	r	0.784	0.769	−0.012	0.010
	*p*	<0.001 *	<0.001 *	0.836	0.860
Accuracy	r		0.868	0.028	−0.022
	*p*		<0.001 *	0.639	0.713
GQS	r			0.005	0.006
	*p*			0.929	0.915
Flesch Reading Ease	r				−0.972
	*p*				<0.001 *

* Spearman correlation analysis, *p* < 0.05 was considered statistically significant. Abbreviations: GQS, Global Quality Score; r, correlation coefficient.

## Data Availability

The raw data supporting the conclusions of this article will be made available by the authors upon request.

## Supplementary Materials

The following supporting information can be downloaded at https://www.mdpi.com/article/10.3390/healthcare14101339/s1, Supplementary Tables: Supplementary Table S1. Modified Brief DISCERN items and scoring criteria used for the evaluation of AI-generated responses; Supplementary Table S2. Post hoc Bonferroni-adjusted pairwise comparisons of Modified Brief DISCERN scores across question categories; Supplementary Table S3. Post hoc Bonferroni-adjusted pairwise comparisons of Accuracy scores across question categories; Supplementary Table S4. Post hoc Bonferroni-adjusted pairwise comparisons of Global Quality Score (GQS) scores across question categories; Supplementary Table S5. Post hoc Bonferroni-adjusted pairwise comparisons of Flesch Reading Ease scores across question categories; Supplementary Table S6. Post hoc Bonferroni-adjusted pairwise comparisons of Flesch-Kincaid Grade Level scores across question categories; Supplementary Table S7. Category-specific correlation analyses between evaluation scores; Supplementary File S1. Complete list of patient-oriented questions included in the study.
