# Supplementary material for: Evaluation of ChatGPT as a Source of Patient-Oriented Information on Gingival Recession
Source: Healthcare (Basel). 2026 May 13;14(10):1339. doi: 10.3390/healthcare14101339 (PMC13205336; doi:10.3390/healthcare14101339)
Supplement: Supplementary file 1 [file healthcare-14-01339-s001.zip › healthcare-4129690-supplementary/healthcare-4129690-Supplementary File S1.pdf]

Supplementary File 1. Complete list of patient-oriented questions included in the study.

T1 – Definition / General Information

T1.01 | What is gingival recession?
T1.02 | Why does the gum move upward?
T1.03 | Does gingival recession mean tooth loss?
T1.04 | Is gingival recession a disease?
T1.05 | Is gingival recession related to aging?
T1.06 | Does gingival recession occur only in older people?
T1.07 | Can gingival recession be stopped?
T1.08 | Can gingival recession be reversed?
T1.09 | Is gingival recession the same as bone loss?
T1.10 | How can gingival recession be noticed?
T1.11 | Is gingival recession an aesthetic problem?
T1.12 | Does everyone with gingival recession always need treatment?
T1.13 | How long does it take for gingival recession to develop?
T1.14 | Can gingival recession occur in children?
T1.15 | Does everyone with gingival recession need surgery?

T2 – Causes / Risk Factors

T2.01 | Can tooth brushing cause gingival recession?
T2.02 | Does using a hard toothbrush lead to gingival recession?
T2.03 | Is brushing teeth too frequently harmful?
T2.04 | Can incorrect brushing technique cause gingival recession?
T2.05 | Does clenching or grinding teeth affect gingival recession?
T2.06 | Can dental calculus cause gingival recession?
T2.07 | Can gingivitis lead to gingival recession over time?
T2.08 | Does periodontitis cause gingival recession?
T2.09 | Do genetic factors affect gingival recession?
T2.10 | Does smoking accelerate gingival recession?
T2.11 | Can gingival recession occur after orthodontic treatment?
T2.12 | Does gingival recession increase with age?
T2.13 | Can gingival recession result from poor oral hygiene?
T2.14 | Does diet influence gingival recession?
T2.15 | Do systemic diseases affect gingival recession?
T2.16 | Can removable dentures cause gingival recession?
T2.17 | Can faulty fillings or crowns cause gingival recession?
T2.18 | Can scaling cause gingival recession?
T2.19 | After scaling my teeth look longer—is this recession?
T2.20 | Does gingival recession increase after scaling?

T3 – Prevention / Protective Measures

T3.01 | Is it possible to prevent gingival recession?
T3.02 | How should I brush my teeth to prevent gingival recession?
T3.03 | Do electric toothbrushes help prevent gingival recession?
T3.04 | Which toothbrush is more suitable for preventing gingival recession?
T3.05 | Which toothpastes are better for gum health?
T3.06 | Does using an oral irrigator prevent gingival recession?
T3.07 | Does dental floss harm or benefit the gums?
T3.08 | Does regular scaling prevent gingival recession?
T3.09 | Do dietary habits affect gingival recession?
T3.10 | Does quitting smoking slow gingival recession?
T3.11 | Does using a night guard help prevent gingival recession?
T3.12 | What should I do when I notice gingival recession?
T3.13 | Does regular dental check-up prevent gingival recession?
T3.14 | If there is a family history of gingival recession, can early prevention help?
T3.15 | Is early detection of gingival recession advantageous?

T4 – Symptoms / Impact on Daily Life

T4.01 | Does gingival recession cause pain?
T4.02 | Does gingival recession cause tooth sensitivity?
T4.03 | Does gingival recession cause discomfort with hot or cold foods?
T4.04 | Can gingival recession cause bad breath?
T4.05 | Does gingival recession affect appearance?
T4.06 | Does gingival recession affect my smile?
T4.07 | Does gingival recession affect speech?
T4.08 | Does food accumulate in areas with gingival recession?
T4.09 | Does gingival recession cause gaps between teeth?
T4.10 | Does gingival recession affect chewing function?
T4.11 | Does gingival recession affect daily life?
T4.12 | Does gingival recession affect self-confidence?
T4.13 | Does gingival recession affect social life?
T4.14 | Should I worry about tooth loss due to gingival recession?
T4.15 | Can gingival recession heal on its own?
T4.16 | How can I tell if gingival recession is progressing?

T5 – Treatment Options

T5.01 | What treatment options are available for gingival recession?
T5.02 | Can gingival recession be treated with laser therapy?
T5.03 | What does grafting mean in gingival recession treatment?
T5.04 | Is graft surgery necessary for every recession?
T5.05 | Can fillings be used to treat gingival recession?
T5.06 | Can pink esthetic procedures correct gingival recession?
T5.07 | Is crown treatment appropriate for gingival recession?

T5.08 | In which cases is non-surgical treatment sufficient?
T5.09 | Is flap surgery performed for gingival recession?
T5.10 | Is root surface debridement effective for gingival recession?
T5.11 | Is graft treatment painful?
T5.12 | Can composite restorations be placed in areas with gingival recession?
T5.13 | Which types of grafts are used for gingival recession?
T5.14 | What is the difference between free gingival grafts and connective tissue grafts?
T5.15 | Is additional treatment required after surgery?
T5.16 | What happens if graft surgery fails?
T5.17 | Are there treatment options other than surgery?
T5.18 | Can biomaterials such as PRF or CGF be used?
T5.19 | Should laser or graft treatment be preferred for gingival recession?
T5.20 | Are sutures placed after graft surgery?
T5.21 | When are sutures removed?
T5.22 | Can gingival recession recur after surgery?
T5.23 | Can graft surgery be repeated if necessary?
T5.24 | What should I pay attention to during the healing period?
T5.25 | Is preparation required before graft surgery?
T5.26 | How long does graft surgery take?
T5.27 | Is hospitalization required for graft surgery?
T5.28 | Is the procedure performed under local anesthesia?
T5.29 | Which dental specialist should I consult for gingival recession?
T5.30 | Will the appearance of my teeth improve after treatment?
T5.31 | Does smoking affect graft success?
T5.32 | Is gingival recession treatment expensive?
T5.33 | Is graft treatment covered by social security insurance?
T5.34 | Can grafts be performed on multiple areas at the same time?
T5.35 | When should I return for follow-up after graft surgery?

T6 – Suitable / Not Suitable

T6.01 | Does every patient with gingival recession require surgical treatment?
T6.02 | Is treatment necessary for mild gingival recession?
T6.03 | What happens if the recession level is not suitable for treatment?
T6.04 | Is tooth sensitivity required to perform graft surgery?
T6.05 | Can grafting be done for purely esthetic reasons?
T6.06 | Can graft surgery be performed in smokers?
T6.07 | I have diabetes—can I undergo graft surgery?
T6.08 | Can gingival recession be treated during pregnancy?
T6.09 | Can graft surgery be performed in patients under 18 years old?
T6.10 | Is graft surgery possible in elderly patients?
T6.11 | Can graft surgery be performed in patients using psychiatric medications?
T6.12 | Can patients taking anticoagulant drugs undergo graft surgery?
T6.13 | Can graft surgery be performed if there is a filling in the affected area?
T6.14 | Can graft surgery be repeated in the same area?
T6.15 | Is general health status important for graft surgery?
T6.16 | Can graft surgery be performed around implants?
T6.17 | Does graft success vary depending on personal factors?

T6.18 | Can patients with limited mouth opening undergo graft surgery?
T6.19 | Is allergy testing required before graft surgery?
T6.20 | Does graft selection change according to recession depth?
T6.21 | Can graft surgery be performed in patients with a history of periodontitis?
T6.22 | Is graft surgery possible if bone loss is present?
T6.23 | Can graft surgery be performed if periodontal disease is active?
T6.24 | Can graft surgery be performed in patients with bruxism?
T6.25 | Can patients with severe gag reflex undergo graft surgery?
T6.26 | Can graft surgery be performed in patients with poor oral hygiene?
T6.27 | Can graft surgery be performed during orthodontic treatment?
T6.28 | Can grafts be performed in areas with crowns or bridges?
T6.29 | Can removable denture users undergo graft surgery?
T6.30 | Is graft surgery recommended for anxious patients?
T6.31 | Are special precautions required for patients with systemic diseases?
T6.32 | Is time off work required after graft surgery?
T6.33 | Is scaling mandatory before graft surgery?
T6.34 | Should early-stage gingival recession be monitored before treatment?
T6.35 | If gingival recession has stopped progressing, is treatment unnecessary?

T7 – Complications / Success Rates

T7.01 | What is the most common complication after graft surgery?
T7.02 | What happens if the graft does not survive?
T7.03 | Is bleeding normal after graft surgery?
T7.04 | Is there a risk of infection after graft surgery?
T7.05 | How long does pain last after graft surgery?
T7.06 | Is bruising around the graft area dangerous?
T7.07 | What happens if graft sutures open?
T7.08 | Is it a problem if sutures are not placed after graft surgery?
T7.09 | Can graft surgery cause poor esthetic outcomes?
T7.10 | Can graft surgery be repeated if unsuccessful?
T7.11 | Do lip movements affect graft healing?
T7.12 | Do jaw movements affect graft healing?
T7.13 | Can numbness occur after graft surgery?
T7.14 | Is tissue difference felt after graft surgery?
T7.15 | Is tissue hardness after graft surgery temporary?
T7.16 | Is recurrence of recession normal after graft surgery?
T7.17 | Is permanent color difference possible after graft surgery?
T7.18 | Can gingival recession accelerate after graft surgery?
T7.19 | Can tooth mobility occur after graft surgery?
T7.20 | Can cold sensitivity develop after graft surgery?
T7.21 | Is facial swelling normal after graft surgery?
T7.22 | How long does gingival swelling last after surgery?
T7.23 | Can tissue breakdown occur after graft surgery?
T7.24 | Can brushing cause tissue loss after graft surgery?
T7.25 | When can graft success be evaluated?
T7.26 | Will esthetic irregularities resolve over time?
T7.27 | What should be done if recession recurs after graft surgery?

T7.28 | Can tissue sagging occur after graft surgery?
T7.29 | Can bad breath occur after graft surgery?
T7.30 | How should the graft area be cleaned postoperatively?
T7.31 | Does smoking cause graft failure?
T7.32 | Can numbness persist after graft surgery?
T7.33 | Can saliva damage sutures?
T7.34 | Is a white layer over the graft area normal?
T7.35 | Is food impaction in the graft area problematic?
T7.36 | When can repeat surgery be performed if graft fails?

T8 – Preprosthetic Period

T7A.01 | Should gingival recession be treated before crown placement?
T7A.02 | Does graft surgery damage crowned teeth?
T7A.03 | When can crowns be placed after graft surgery?
T7A.04 | Can graft surgery be performed for recession under a crown?
T7A.05 | How long should one wait before taking impressions after graft surgery?
T7A.06 | Does placing crowns without grafting affect long-term success?

T9 – Postprosthetic Period

T7B.01 | Are existing crowns replaced after graft surgery?
T7B.02 | What happens if the crown margin pressures the graft area?
T7B.03 | Should I be careful while eating with my prosthesis after graft surgery?
T7B.04 | Is prosthesis re-adjusted after graft surgery?
T7B.05 | Is using a temporary prosthesis harmful after graft surgery?
T7B.06 | What should be done if the crown does not fit the gingiva after surgery?
T7B.07 | Does grafting over prosthetic areas improve esthetics?

T10 – Early Postop (0 – 7 days)

T8.01 | How long does pain last after surgery?
T8.02 | Is swelling normal after graft surgery?
T8.03 | What should I do if bleeding occurs?
T8.04 | Is ice application necessary after surgery?
T8.05 | Should hot foods be avoided on the first day?
T8.06 | Does chewing on the graft area cause damage?
T8.07 | Is tooth brushing harmful in the early postoperative period?
T8.08 | When are sutures removed after surgery?
T8.09 | Should the graft area be brushed?
T8.10 | Does brushing sutures harm graft healing?
T8.11 | Should mouthwash be used after surgery?
T8.12 | Does spitting blood affect graft success?
T8.13 | Does smoking impair graft healing?

T8.14 | Are antibiotics necessary after graft surgery?
T8.15 | How long should painkillers be used?
T8.16 | What foods can I eat after surgery?
T8.17 | Is soft diet required during the first week?
T8.18 | What happens if the graft area is touched?
T8.19 | Can I exercise during the first week?
T8.20 | When can mouth rinsing begin?
T8.21 | What should I do if the graft moves?
T8.22 | Does a fallen graft require repeat surgery?
T8.23 | How should sutured areas be protected?
T8.24 | Is excessive talking harmful after surgery?
T8.25 | Is fever normal after graft surgery?

T11 – Late Postop (1 week–6 months)

T9.01 | How long does complete healing take after graft surgery?
T9.02 | Is color change in the graft area normal?
T9.03 | Is graft shrinkage normal after one month?
T9.04 | Is white appearance of the graft area problematic?
T9.05 | What should be done if recession persists after two months?
T9.06 | Is persistent sensitivity a sign of graft failure?
T9.07 | Is color mismatch between graft and gingiva normal?
T9.08 | Does the graft adapt to surrounding tissue over time?
T9.09 | Is hardness of the graft area normal?
T9.10 | When does the final appearance stabilize?
T9.11 | How can I tell if the graft has failed?
T9.12 | What should be done if recession recurs after six months?
T9.13 | Is long-term recurrence possible after graft surgery?
T9.14 | Can esthetic corrections be made if the result is unsatisfactory?
T9.15 | Is smile asymmetry permanent after graft surgery?
T9.16 | How should brushing be performed after healing?
T9.17 | Which toothpastes are recommended after graft surgery?
T9.18 | What happens if the graft area is brushed too hard?
T9.19 | What is evaluated during follow-up visits?
T9.20 | Is feeling graft enlargement normal?
T9.21 | How often should follow-up visits be scheduled?
T9.22 | Can scaling damage the graft area?
T9.23 | Can crowns be placed six months after graft surgery?
T9.24 | Can orthodontic treatment be performed over grafted areas?
T9.25 | Is tissue thickening after graft surgery beneficial?
T9.26 | How does the clinician determine graft adequacy?

T12 – What Happens If Left Untreated?

T10.01 | What happens if gingival recession is left untreated?
T10.02 | Can gingival recession stop spontaneously without treatment?

T10.03 | Can untreated gingival recession lead to tooth loss?
T10.04 | Does untreated gingival recession cause bone loss?
T10.05 | Does tooth sensitivity increase if untreated?
T10.06 | Does gingival recession become painful over time?
T10.07 | Does untreated recession worsen esthetics?
T10.08 | Do spaces between teeth increase?
T10.09 | Does gingival recession cause bad breath?
T10.10 | Can gingival recession affect speech?
T10.11 | Does gingival recession impair chewing function?
T10.12 | Can untreated recession harm neighboring teeth?
T10.13 | Can gingival recession progress to tooth loss?
T10.14 | Does untreated recession affect orthodontic treatment?
T10.15 | Does gingival recession complicate prosthetic planning?
T10.16 | Can gingival recession prevent future implant placement?
T10.17 | Does delaying treatment reduce success rates?
T10.18 | Does delaying treatment increase costs?
T10.19 | Does early intervention simplify treatment?
T10.20 | Can untreated gingival recession have systemic effects?
T10.21 | Does gingival recession cause psychological distress?
T10.22 | Does gingival recession affect facial esthetics?

T13 – Psychological / Social Effects

T11.01 | Is it normal for gingival recession to affect self-confidence?
T11.02 | Do individuals with gingival recession avoid social settings?
T11.03 | Do esthetic concerns influence treatment decisions?
T11.04 | Do patients cover their mouth while smiling due to recession?
T11.05 | Does gingival recession cause anxiety during speaking?
T11.06 | Does gingival recession affect work performance?
T11.07 | Does gingival recession alter facial expression?
T11.08 | Can gingival recession affect mood?
T11.09 | Are individuals with gingival recession more prone to depression?
T11.10 | Does gingival recession cause shyness in relationships?
T11.11 | Does gingival recession have greater psychological impact in younger individuals?
T11.12 | Is smile design reasonable for gingival recession?
T11.13 | Does social media increase awareness of gingival esthetics?
T11.14 | Does gingival recession increase interest in esthetic dentistry?
T11.15 | Does self-confidence improve after gingival treatment?

T14 – Information Sources and Artificial Intelligence Reliability

T12.01 | Do AI systems provide accurate information about gingival recession?
T12.02 | Do AI systems such as ChatGPT cite their sources?
T12.03 | Can AI responses without references be trusted?
T12.04 | Do AI systems always provide up-to-date information?
T12.05 | Can AI systems provide incorrect information?

T12.06 | Can AI systems replace dentists?
T12.07 | Are AI systems unbiased or do they provide one-sided information?
T12.08 | Is it normal for AI systems to give different answers to the same question?
T12.09 | Can AI systems provide personalized recommendations?
T12.10 | Is it risky to act on AI information without consulting a dentist?
T12.11 | Can AI systems assist in decision-making for gingival recession treatment?
T12.12 | In which situations can AI systems be misleading?
T12.13 | How can I recognize unreliable AI-generated information?
T12.14 | Do AI systems rely on scientific journals as information sources?
T12.15 | Can AI systems give false hope to patients?
