# Supplementary material for: Evaluation of ChatGPT as a Source of Patient-Oriented Information on Gingival Recession
Source: Healthcare (Basel). 2026 May 13;14(10):1339. doi: 10.3390/healthcare14101339 (PMC13205336; doi:10.3390/healthcare14101339)
Supplement: Supplementary file 1 [file healthcare-14-01339-s001.zip › healthcare-4129690-supplementary/healthcare-4129690-supplementary Tables.pdf]

SUPPLEMENTARY TABLES

Supplementary Table S1. Modified Brief DISCERN items and scoring criteria used for the evaluation of AI-generated responses.

Order	Question Title	What is being evaluated?
D1	Is the information reliable?	Is the information scientifically based? Is there any misleading or inaccurate information?
D2	Is the information up-to-date?	Is the content aligned with current guidelines, standards, or scientific developments?
D3	Is the information unbiased?	Is the information presented in a balanced manner? Does it favor certain treatments while excluding others?
D4	Are risks and benefits stated?	Have both the pros and cons of gingival recession treatment options, etc. been discussed?
D5	Does it help you decide?	Does the information help patients or readers make informed decisions?

* Items were scored on a 5-point Likert scale (1 = not present, 5 = completely fulfilled); higher total scores indicate higher information quality. These five items were adapted from the principal conceptual domains represented in the Brief DISCERN framework to support structured evaluation of AI-generated patient information in the present study; the adapted form was used as a study-specific operational derivative rather than as a newly validated replacement for the original Brief DISCERN instrument.

Supplementary Table S2. Post hoc Bonferroni-adjusted pairwise comparisons of modified Brief DISCERN scores across question categories.

	**p value***
What Happens If Left Untreated? < Prevention / Protective Measures	0.005
What Happens If Left Untreated? < Symptoms / Daily Life Impacts	<0.001
What Happens If Left Untreated? < Information Sources / AI Reliability	<0.001
Psychological / Social Effects < Symptoms / Daily Life Impacts	0.003
Psychological / Social Effects < Information Sources / AI Reliability	0.001
Treatment Options < Symptoms / Daily Life Impacts	0.001
Treatment Options < Information Sources / AI Reliability	<0.001
Complications / Success Rates < Symptoms / Daily Life Impacts	0.006
Complications / Success Rates < Information Sources / AI Reliability	0.001
Late Post-op (1 week–6 months) < Information Sources / AI Reliability	0.015
Suitable / Not Suitable < Symptoms / Daily Life Impacts	0.038
Suitable / Not Suitable < Information Sources / AI Reliability	0.008

*Data are presented as Bonferroni-adjusted post hoc pairwise comparisons following Kruskal–Wallis analysis.

$p < 0.05$ was considered statistically significant.

Supplementary Table S3. Post hoc Bonferroni-adjusted pairwise comparisons of Accuracy scores across question categories.

	**p value***
What Happens If Left Untreated? < Information Sources / AI Reliability	<0.001
Suitable / Not Suitable < Information Sources / AI Reliability	<0.001
Treatment Options < Information Sources / AI Reliability	<0.001
Causes / Risk Factors < Information Sources / AI Reliability	<0.001
Psychological / Social Effects < Information Sources / AI Reliability	<0.001
Complications / Success Rates < Information Sources / AI Reliability	<0.001
Late Post-op (1 week–6 months) < Information Sources / AI Reliability	<0.001
Prevention / Protective Measures < Information Sources / AI Reliability	0.008
Definition / General Information < Information Sources / AI Reliability	0.032
Early Post-op (0–7 days) < Information Sources / AI Reliability	0.047

* Data are presented as Bonferroni-adjusted post hoc pairwise comparisons following Kruskal–Wallis analysis.

$p < 0.05$ was considered statistically significant.

Supplementary Table S4. Post hoc Bonferroni-adjusted pairwise comparisons of Global Quality Scale (GQS) scores across question categories.

	**p value***
What Happens If Left Untreated? < Information Sources / AI Reliability	<0.001
Complications / Success Rates < Symptoms / Daily Life Impacts	0.026
Complications / Success Rates < Information Sources / AI Reliability	<0.001
Postprosthetic Period < Information Sources / AI Reliability	0.027
Suitable / Not Suitable < Information Sources / AI Reliability	<0.001
Treatment Options < Information Sources / AI Reliability	<0.001
Causes / Risk Factors < Information Sources / AI Reliability	0.002
Late Post-op (1 week–6 months) < Information Sources / AI Reliability	0.025

* Data are presented as Bonferroni-adjusted post hoc pairwise comparisons following Kruskal–Wallis analysis.

$p < 0.05$ was considered statistically significant.

Supplementary Table S5. Post hoc Bonferroni-adjusted pairwise comparisons of Flesch Reading Ease scores across question categories.

	P*
Treatment Options < Prevention / Protective Measures	0.017

* Data are presented as Bonferroni-adjusted post hoc pairwise comparisons following Kruskal–Wallis analysis.

$p < 0.05$ was considered statistically significant.

Supplementary Table S6. Post hoc Bonferroni-adjusted pairwise comparisons of Flesch–Kincaid Grade Level scores across question categories.

	p value*
Prevention / Protective Measures < Treatment Options	0.010
Complications / Success Rates < Treatment Options	0.033

*Data are presented as Bonferroni-adjusted post hoc pairwise comparisons following Kruskal–Wallis analysis.

$p < 0.05$ was considered statistically significant.

Supplementary Table S7. Category-specific correlation analyses between evaluation scores.

			Accuracy	GQS	FLESCH Ease	FLESCH Kincaid Grade Level
Definition / General Information	DISCERN	r	0.604	0.853	-0.415†	0.259†
		p	0.017*	<0.001*	0.124	0.352
	Accuracy	r		0.775	-0.458	0.393
		p		0.001*	0.086	0.147
	GQS	r			-0.352	0.113
		p			0.198	0.689
	FLESCH Reading Ease	r				-0.926†
		p				<0.001*
Causes/Risk Factors	DISCERN	r	0.882	0.882	0.158	-0.214†
		p	<0.001*	<0.001*	0.505	0.366
	Accuracy	r		1.000	0.312	-0.383
		p		<0.001*	0.181	0.096
	GQS	r			0.312	-0.383
		p			0.181	0.096
	FLESCH Reading Ease	r				-0.900
		p				<0.001*
Prevention / Protective Measures	DISCERN	r	0.811	0.811	0.405	-0.297
		p	<0.001*	<0.001*	0.135	0.283
	Accuracy	r		1.000	0.378	-0.252
		p		<0.001*	0.165	0.364

	GQS	r			0.378	-0.252
		p			0.165	0.364
	FLESCH Reading Ease	r				-0.839†
		p				<0.001*
Symptoms / Daily Life Impacts	DISCERN	r	0.231	0.231	0.382	-0.366
		p	0.390	0.390	0.144	0.163
	Accuracy	r		1.000	-0.174	0.226
		p		<0.001*	0.520	0.399
	GQS	r			-0.174	0.226
		p			0.520	0.399
	FLESCH Reading Ease	r				-0.906
		p				<0.001*
Treatment Options	DISCERN	r	0.746	0.746	0.028	-0.029
		p	<0.001*	<0.001*	0.874	0.867
	Accuracy	r		1.000	0.123	-0.111
		p		<0.001*	0.482	0.526
	GQS	r			0.123	-0.111
		p			0.482	0.526
	FLESCH Reading Ease	r				-0.988
		p				<0.001*
Suitable / Not Suitable	DISCERN	r	0.788	0.782	-0.116	0.133
		p	<0.001*	<0.001*	0.508	0.446
	Accuracy	r		0.893	-0.082	0.084
		p		<0.001*	0.640	0.630
	GQS	r			-0.137	0.165
		p			0.433	0.343
		r				-0.989

	FLESCH Reading Ease	p				<0.001*
Complications / Success Rates	DISCERN	r	0.766	0.715	-0.069†	0.143†
		p	<0.001*	<0.001*	0.896	0.787
	Accuracy	r		0.615	0.225	-0.166
		p		<0.001*	0.187	0.332
	GQS	r			0.255	-0.186
		p			0.133	0.278
	FLESCH Reading Ease	r				-0.991†
		p				<0.001*
Preprosthetic Period	DISCERN	r	0.853	0.804	-0.313	0.379
		p	0.031*	0.054	0.545	0.459
	Accuracy	r		0.707	0.105	1.000
		p		0.116	0.843	<0.001*
	GQS	r			-0.396	0.503
		p			0.437	0.310
	FLESCH Reading Ease	r				-0.985
		p				<0.001*
Postprosthetic Period	DISCERN	r	0.661	0.322	0.600†	-0.504†
		p	0.106	0.481	0.154	0.248
	Accuracy	r		0.548	0.144	-0.433
		p		0.203	0.758	0.332
	GQS	r			1.000	1.000
		p			<0.001*	<0.001*
	FLESCH Reading Ease	r				-0.924†
		p				0.003*
	DISCERN	r	0.696	0.813	-0.222	0.241

Early Post-op (0–7 days)		p	<0.001*	<0.001*	0.287	0.246
	Accuracy	r		0.688	-0.085	0.091
		p		<0.001*	0.688	0.665
	GQS	r			0.023*	1.000
		p			0.914	<0.001*
	FLESCH Reading Ease	r				-0.978†
		p				<0.001*
Late Post-op (1 week–6 months)	DISCERN	r	0.847	0.847	-0.328	0.332
		p	<0.001*	<0.001*	0.102	0.098
	Accuracy	r		1.000	-0.242	0.242
		p		<0.001*	0.234	0.233
	GQS	r			-0.242	0.242
		p			0.234	0.233
	FLESCH Reading Ease	r				-0.981†
		p				<0.001*
What Happens If Left Untreated?	DISCERN	r	0.739	0.739	-0.186	0.149
		p	<0.001*	<0.001*	0.407	0.507
	Accuracy	r		1.000	-0.163	0.145
		p		<0.001*	0.470	0.518
	GQS	r			-0.163	0.145
		p			0.470	0.518
	FLESCH Reading Ease	r				-0.931†
		p				<0.001*
Psychological / Social Effects	DISCERN	r	0.740	0.925	0.280	-0.327
		p	0.002*	<0.001*	0.312	0.235

	Accuracy	r		0.764	0.557	-0.588
		p		0.001*	0.031	0.021
	GQS	r			0.410	-0.441
		p			0.129	0.100
	FLESCH Reading Ease	r				-0.977†
		p				<0.001*
Information Sources / AI Reliability	DISCERN	r	0.826	0.711	0.471	-0.579†
		p	<0.001*	0.003*	0.077	0.024
	Accuracy	r		0.500	0.394	-0.442
		p		0.058	0.146	0.099
	GQS	r			0.329	-0.278
		p			0.232	0.315
	FLESCH Reading Ease	r				-0.970
		p				<0.001*

*Spearman correlation analysis, p<0.05 was considered statistically significant, † Indicates negative correlation.

Abbreviations: DISCERN, modified Brief DISCERN score; GQS, Global Quality Scale; r, correlation coefficient.
